# A Landscape Analysis of Offering HIV Testing Services Within Family Planning Service Delivery

**DOI:** 10.3389/frph.2021.657728

**Published:** 2021-05-26

**Authors:** Alison L. Drake, Caitlin Quinn, Nancy Kidula, Euphemia Sibanda, Petrus Steyn, Magdalena Barr-DiChiara, Muhammad S. Jamil, Michelle Rodolph, Mary E. Gaffield, James Kiarie, Rachel Baggaley, Cheryl Johnson

**Affiliations:** ^1^Department of Global Health, University of Washington, Seattle, WA, United States; ^2^Department of Global HIV, Hepatitis and Sexually Transmitted Infections Programmes, World Health Organization, Geneva, Switzerland; ^3^WHO Regional Office for Africa, Intercounty Support Team for Eastern and Southern Africa, Harare, Zimbabwe; ^4^Centre for Sexual Health and HIV/AIDS Research, Harare, Zimbabwe; ^5^The United Nations Development Programme/UNFPA/UNICEF/WHO/World Bank Special Programme of Research, Development and Research Training in Human Reproduction (HRP Research), Geneva, Switzerland

**Keywords:** HIV testing services, family planning, sexual and reproctive health, service delivery, service integration

## Abstract

**Introduction:** Offering HIV testing services (HTS) within sexual and reproductive health (SRH) services is a priority, especially for women who have a substantial risk. To reach women with HIV who do not know their status and prevent mother-to-child HIV transmission, the World Health Organization (WHO) recommends routinely offering HTS as part of family planning (FP) service delivery in high HIV burden settings. We conducted a landscape analysis to assess HTS uptake and HIV positivity in the context of FP/SRH services.

**Assessment of Research and Programs:** We searched records from PubMed, four gray literature databases, and 13 organization websites, and emailed 24 organizations for data on HTS in FP/SRH services. We also obtained data from International Planned Parenthood Federation (IPPF) affiliates in Eswatini, Kenya, Lesotho, Malawi, Namibia, Uganda, Zambia, and Zimbabwe. Unique programs/studies from records were included if they provided data on, or barriers/facilitators to, offering HTS in FP/SRH. Overall, 2,197 records were screened and 12 unique programs/studies were eligible, including 10 from sub-Saharan Africa. Four reported on co-delivery of SRH services (including FP), with reported HTS uptake between 17 and 94%. Six reported data on HTS in FP services: four among general FP clients; one among couples; and one among female sex workers, adolescent girls, and young women. Two of the six reported HTS uptake >50% (51%, 419/814 Kenya; 63%, 5,930/9,439 Uganda), with positivity rates of 2% and 4.1%, respectively. Uptake was low (8%, 74/969 Kenya) in the one FP program offering pre-exposure prophylaxis. In the IPPF program, seven countries reported HTS uptake in FP services and ranged from 4% in Eswatini to 90% in Lesotho; between 0.6% (Uganda) and 8% (Eswatini) of those tested were HIV positive.

**Implications:** Data on providing HTS in FP/SRH service delivery were sparse and HTS uptake varied widely across programs.

**Actionable Recommendations:** As countries expand HTS in FP/SRH appropriate to epidemiology, they should ensure data are reported and monitored for progress and impact.

## Introduction

Women of reproductive age have disproportionately high risks of HIV in sub-Saharan Africa (1, 2). Increased efforts to identify women with HIV and link them to care and treatment are imperative to reach the UN 95-95-95 fast track targets (3). Recent data from the ECHO trial conducted among women seeking family planning (FP) services in high HIV burden settings in Africa demonstrated high HIV incidence of 3.8 per 100 woman-years (4). These results highlight the need for integrated HIV service delivery among women who seek FP for pregnancy prevention. Integrating HIV and sexual reproductive health (SRH) has long been considered a priority and routinely offering testing in antenatal care clinics has been widely accepted with high uptake (5). To reach the women with unknown HIV status and prevent mother-to-child HIV transmission, routine offer of HIV testing services (HTS) for women seeking FP services in high HIV burden countries is also recommended by the World Health Organization (WHO) (6, 7). However, there has been much less global commitment and focus on HIV testing within the context of FP and SRH services. Failure to test women seeking FP services represents a missed opportunity to identify women with undiagnosed HIV who can be linked to antiretroviral treatment (ART), re-engage women who have been previously diagnosed with HIV and are not on ART, identify HIV-negative women who could benefit from a range of HIV prevention choices [including pre-exposure prophylaxis (PrEP)], and provide the opportunity to deliver partner services for those with HIV.

Despite the recognition of benefits of improving HIV-SRH linkages for many years (8, 9) and development of resources to support integrated services, little real-world progress has been made beyond some efforts to integrate FP into postnatal care in prevention of mother-to-child transmission (PMTCT) programs and offering FP for women receiving HIV care and treatment (10–13). Providing HTS in FP settings as a specific approach to integrated service delivery has received considerably less attention.

A recent systematic review suggested that integration of HTS in FP settings was feasible and showed potential to improve client satisfaction with services (14). However, evidence was limited to data from six comparative studies conducted in four countries (Kenya, Eswatini, Uganda, and USA) (14). In order to examine country implementation of HTS in routine FP service delivery, we conducted a landscape analysis to assess HTS uptake and HIV positivity in the context of FP service delivery using reports from research and programs, as well as programmatic experiences. We highlight approaches to provide HTS within FP/SRH service delivery to inform implementation.

## Assessment of Research and Programs

### Overview and Inclusion Criteria

A review of comparative studies on integrating HTS into FP was previously conducted (14); we sought to analyze data from non-comparative studies excluded from this review and data published after the review was conducted. Comparative studies from the prior review were excluded from this analysis in order to focus on real-world program implementation that differs from controlled environments. We obtained and reviewed a list of references identified as relevant but excluded in the prior review due to lack of a comparison (i.e., intervention) group, by contacting authors (14). We searched PubMed to capture articles published after the prior review as well as gray literature databases. No geographical restrictions were applied to the references from the prior review or database searches. We also reviewed organizational websites [including government and non-government organizations (NGOs)] known to implement or research HIV and FP/SRH in sub-Saharan Africa and emailed contacts at these organizations to request study or program data on HTS in FP/SRH services. In addition, we conducted semi-structured phone interviews with program managers from International Planned Parenthood Federation (IPPF) member associations in eight countries: Eswatini, Kenya, Lesotho, Malawi, Namibia, Uganda, Zambia, and Zimbabwe. These countries were selected for specific inquiry as they have been identified as priority countries based on their prevalence of HIV in women of reproductive age and contraceptive prevalence rate <67% (15).

We assessed the proportion of women of reproductive age (age 15–49) who were offered HTS, HTS uptake (defined as providing tests after they are offered), and HIV test positivity as primary outcomes in FP programs. Programs, reports, and other data were included in the review if they (1) described offering HTS into FP services, including offering HTS to women of reproductive age (age 15–49) seeking FP or SRH through clinic- or community-based service delivery, and (2) measured one or more of the primary outcomes or included qualitative perspectives on offering HTS with FP alone or FP in conjunction with other SRH services. Records were excluded if data represented household or community surveys among a general population rather than individuals offered HTS in FP/SRH program service delivery. There were no language restrictions; however, only English terms were used in the search.

### Search Strategy

We used a keyword search in PubMed and four gray literature electronic databases, including Think Tank Search, Gray Literature Report, Open Gray, and Union of International Associations IGO. Key words were “HIV” AND “contraception,” “HIV” AND “family planning,” “HIV” AND “birth control,” and “HIV” AND “integration.” For databases that accept MeSH terms, we used the following MeSH terms: ((“HIV Infections/diagnosis”[Mesh] OR “AIDS Serodiagnosis”[Mesh]) OR (“Diagnostic Tests, Routine”[Mesh] OR “Mass Screening”[Mesh] OR “testing”[tiab]) AND (“Contraception”[Mesh] OR “FP” OR “birth control”[tiab])). PubMed databases were searched through from June 21, 2017 to March 20, 2020. Gray literature searches were performed between May 15 and 24, 2019. A snowball approach was used to search websites, in which new organizations identified from searching the initial list were added to the search. The websites included in the search included Center for Strategic and International Studies (CSIS), Family Planning 2020, FHI 360, Frontline AIDS, Integra Initiative, International Planned Parenthood Federation (IPPF), JHPIEGO, MEASURE Evaluation, PATH, Population Council/The Evidence Project, Sexual and Reproductive Health & HIV Linkages (SRH & HIV Linkages), and United Nations Population Fund (UNFPA). We searched websites using “HIV” and each of the following other terms, individually: “contraception,” “family planning,” “birth control,” and “integration.”

We directly contacted individuals representing government and non-government organizations (NGOs) though email and requested any relevant documents or reports on service delivery of integrating HTS into FP, with a maximum of three reminders to prompt a reply. Individuals could also refer the research team to other contacts. IPPF program managers were invited to participate in a semi-structured phone interview with the research team to discuss data and experiences of implementing integrated programs. Documents and reports from websites and contacts were collected through December 16, 2019.

Titles, abstracts, data summaries, and reports were evaluated for inclusion in the full-text review by a single reviewer. Relevant records were selected for full-text review, and data were extracted independently by one reviewer using a standardized extraction form. We used the following definitions in our analysis to guide decisions on eligibility for inclusion and abstraction of outcomes:

- **FP services:** Health care programs or services designed to assist individuals in preventing or delaying pregnancy, including counseling, referral, dispensing, providing, or removing FP/contraceptive methods.
- **HIV testing services (HTS), including HIV self-testing:** Execution of HIV test procedures, including pre-test information and post-test counseling. We also aimed to abstract data from programs on linkage to HIV prevention, treatment and care services and other clinical and support services.
- **Sexual and reproductive health (SRH) services:** SRH care includes providing antenatal, perinatal, postpartum, and newborn care; FP, fertility, and abortion services; sexually transmitted infections (STIs) screening and treatment, including HIV, reproductive tract infections, cervical cancer, and other gynecological morbidities; and counseling on sexuality (16).
- **Integration:** Integration was defined as the provision of HTS alongside or within FP programs or services (i.e., co-located and/or sharing services and resources) but excludes the provision of FP services within HIV prevention, treatment, and care programs.
- **Social Harms:** Any intended or unintended cause of physical, economic, emotional, or psychosocial injury or hurt from one person to another, a person to themselves, or an institution to a person, occurring before, during, or after testing for HIV (17).

### Analysis

We refer to all data, reported from studies and programs, as program data for simplicity and analyzed the program as the unit of analysis. We classified HTS uptake, among those offered as high (>85%), moderate (50–85%), and low (<50%). HIV positivity was reported if available and was calculated among those who were tested for HIV, excluding those offered and not tested. We used data from UNAIDS from the study period for HIV prevalence and calculated treatment-adjusted prevalence by combining HIV prevalence with population data from World Bank (18, 19). To estimate the expected HTS positivity, we also calculated the treatment-adjusted prevalence (20), which removes the number of PLHIV who are on ART from HIV prevalence and population estimates to determine the expected HTS positivity among those receiving HTS. Due to the heterogeneity in approaches to integrate HTS within FP/SRH service delivery, we did not pool results.

The semi-structured phone interviews with IPPF member association managers were conducted by one interviewer and transcribed during the call. The interviewer contacted respondents by email after the interview for any required clarifications and further collection of programmatic data. We analyzed the qualitative data by organizing responses into conceptual categories and tracking emerging themes from the data. Representative quotes were extracted from the interview notes and organized by themes and sub-themes. Summary text from other included studies that address perspectives on successes and challenges of HIV/FP integration was also extracted and incorporated into the thematic table to further draw out salient themes and experiences related to integration.

### Results

**Figure 1:**
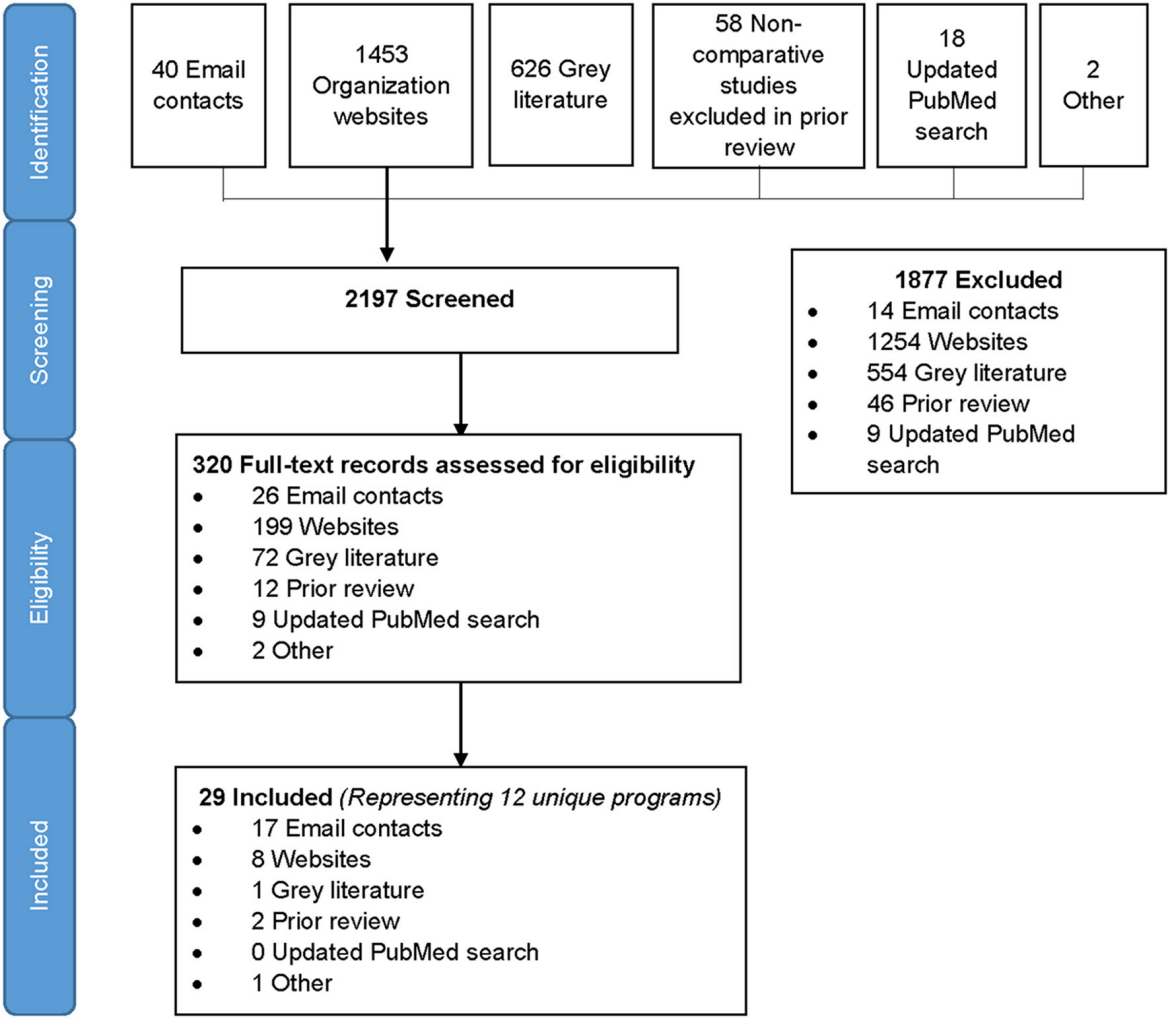
Diagram of record selection.

We identified 2,197 records in the search, of which 1,453 were from organizational websites, 626 from online gray literature databases, 58 from the prior review, 40 from direct email contacts, 18 from PubMed, and 2 others that the team was aware of (Figure 1). After screening titles and abstracts, 320 full-text records were assessed for eligibility and 29 records met eligibility criteria for inclusion. Included records spanned between 2010 and 2019. The 29 eligible records represented 12 unique programs included in the analysis (21–57) (Figure 1). All data were reported or extracted from programs, with the exception of one pilot study of a couples intervention (40). Two-thirds of programs (8/12) reported cross-sectional program data, two programs reported data at different time points (multiple records from the same program) using cross-sectional and pre–post-program reports, one reported pre- and post-program data only, and one was longitudinal. In addition, three programs included qualitative data on program implementation. The majority (10/12) of programs were conducted in sub-Saharan Africa, eight in one or more of the priority countries (Table 1). One SRH program was conducted in multiple locations (four countries in sub-Saharan Africa, India, and Tunisia) (44–46) and two programs were delivered in the United States (*n* = 2) (22, 39). Due to the small number of records outside of sub-Saharan Africa, we summarized results within and outside sub-Saharan Africa separately. Among 10 programs from sub-Saharan Africa, we identified six that were focused on offering HTS within the context of FP service delivery and four within broader SRH programs that included FP.

**Table 1:** Characteristics of included programs/studies from sub-Saharan African countries, by population.

**References**	**Location**	**Target population**	**Time period**	Adult HIV prevalence (95% CI)^**^	Treatment-adjusted prevalence^*^	**Study design**	**Description of approach to offering HTS**
Becker et al. (40)	Malawi (home-based)	Male–female married/unioned couples	2009	10.1 (8.9–11.1)	7.8%	Pre–post	**HTS + FP:** Co-delivery of couples HTS and couples FP on-site in households. Men only offered services if women received services independently (HTS, FP or both) (*N* = 180 couples)
Eastment et al. (35)	Mombasa, Kenya (facility)	New FP clients (female implied)	2016	5.1 (4.4–5.9)	1.4%	Cross-sectional (Review of service delivery statistics)	**HTS + FP:** Measured on-site HTS in a random sample (*n* = 58) FP clinics over 3 months
Mugwanya et al. N.D. and Personal communication (Pintye, Jillian) (24, 25)^a^	Western Kenya (facility)	Female FP clients, including AGYW	2017–2018	5.0 (4.4–5.8)	1.3%	Longitudinal	**HTS + FP:** HTS offered on-site in 8 high-volume FP clinics via a PrEP implementation program
Tassi (56)	Uganda	FP clients (sex not specified)	July–Sept 2018	6.2 (5.8–6.9) 22.9 (21.6–24.8) 26.6 (25.1–28.2)	1.8% 15.1% 18.3%	Cross-sectional (Review of service delivery statistics)	**HTS + FP:** Co-delivery of HTS and FP (on-site and referral) in majority of government facilities in Uganda. Data of HTS among FP clients were provided from 49 selected facilities
SRH & HIV Linkages (21, 42, 44, 58, 59)^b^	All			22.7 (20.8–24.0)	11.9%		**HTS + SRH: Comprehensive on-site co-delivery of SRH/HIV “**Linkages Project” HTS, ART, VMMC scaled-up through partnerships with civil service organizations.
	Lesotho (facility)	Male and female SRH clients; also adolescents, survivors of gender-based violence, FSW, and people with HIV	2012–2013	22.6 (20.9–24.0)	10.2%	Pre–post	HTS, ART, VMMC scaled-up through partnerships with civil service organizations.
	Eswatini (facility)	Female SRH clients	2011–2013	2.3 (1.9–2.8)	0.8%	Cross-sectional (Review of service delivery statistics; patient and provider satisfaction surveys)	5 centers; one-stop shop delivery enhanced by peer mentorship for HCW
	Botswana (facility)	Female SRH clients	2012–2014	5.2 (4.6–5.6)	2.7%	Pre–post	ART, FP, STIs, CaCx screening in 9 pilot sites; one-stop shop delivery enhanced by training and technical support on integration, task-shifting and task-sharing, NGO partnerships.
		Male and female SRH clients	2015	1.2 (0.9–1.7)	1.0%	Cross-sectional (interviews with patients, providers and policymakers)	9 sites using “kiosk” (services by single HCW in same room), “supermarket” (services in multiple rooms by different HCW at large clinics), or “mall” (referral to different rooms within same facility for different services by different HCW in hospitals) models. FP registers updated with HTS and youth-friendly campaign launched.
	Togo (facility)	Male and female SRH clients	Not reported	1.2 (0.9–1.7)	1.1%	Cross-sectional (Service delivery statistics; interviews with patients, providers and policymakers)	Enhanced training providers on SRH and HIV integration
Personal communication (JHPIEGO) (41, 43)^c^	Tanzania (community)	Female SRH clients who were FSW, out of school AGYW, or other hotspot female populations	2014–2017	10.8 (8.6–13.4)	10.4%	Cross-sectional (Review of service delivery statistics)	**HTS + FP:** Co-delivery of HTS and FP with HIV prevention and linkage to ART on-site for key and vulnerable populations. “Sauti Project”
Chukwujekwu et al. and GHAIN report, N.D. (34, 37)^d^	Nigeria (facility)	FP clients (sex not specified)	All	27.1 (25.4–28.8)	1.9%	–	**HTS + FP:** HTS and FP delivered through one-stop shop (FP providers provide both FP and HTS during same visit) and referral-based models (FP providers offer FP and HIV counseling only and refer clients to co-located HTS). Included tools for HIV-FP integration, provider training and supportive supervision. “Global HIV/AIDS Initiative Nigeria (GHAIN)”
			2007–2009	4.8 (4.2–5.6)	1.2%	Pre–post	71 public health facilities.
			2007–2011	23.1 (21.5–25.0)	10.3%	Review of service delivery statistics	141 public health facilities.
Lafort et al. International Centre for Reproductive Health (ICRH) (33)^e^	Tete, Mozambique (community)	FSW	2004–2009	12.7 (11.7–13.8)	2.4%	Cross-sectional (Service delivery statistics; key informant interviews; FGDs)	**HTS + SRH:** Co-delivery of FP, STI, and HTS on-site at a night clinic (4–10 PM) for FSW with free services and expanded peer outreach activities. “Diagonal Interventions to Fast-Forward Reproductive Health (DIFFER)”
International Planned Parenthood Federation (IPPF) (49–55, 57)^f^	All (facility and community)	Male and female SRH clients	2019	9.5 (8.8–10.1)	2.3%	Cross-sectional (Program data & interview with program managers)	**HTS + SRH:** Comprehensive co-delivery of SRH and HIV services in static and mobile clinics (on-site and referral).
	Eswatini (FLAS)		–	12.1 (11.3–13.1)	2.0%	–	HIV services include HTS, ART, VMMC, and PEP
	Kenya (FHOK)	–	–	6.1 (5.7–6.8)	1.0%	(Interview only)	HTS for HIV services and referral for ART
	Lesotho (LPPA)		–	13.4 (11.8–15.3)	2.6%	–	HIV services include HTS, ART, and VMMC; clinics include men's and youth clinics.
	Namibia (NAPPA)	Focus on youth (10–24 years)	–	5.1 (4.5–5.5)	3.7%	–	HIV services include HTS, ART, and VMMC; all clinics youth friendly.
	Malawi (FPAM)	Focus on youth (10–24 years)	–	10.1 (8.9–11.1)	7.8%	–	HIV services include HTS, ART, and VMMC; all clinics youth friendly.
	Zambia (PPAZ)	–	–	5.1 (4.4–5.9)	1.4%	–	Co-delivery of FP (and related SRH) with HTS in 3 static clinics, 11 mobile units, and 10 community-based services (on-site and referral). Referral for ART and PMTCT.
	Uganda (RHU)	–	–	5.0 (4.4–5.8)	1.3%	–	Mainly focused on reaching key populations. Offer of HTS is routine in every interface with client.
	Zimbabwe (ZNFPC)	–	–	6.2 (5.8–6.9)	1.8%	–	Co-delivery of FP (and related SRH) with HTS in 10 static and mobile clinics (plus a few youth-focused centers) (on-site and referral).
Plotkin et al. (23)^g^	Tanzania (facility)	Female SRH clients	2010–2013	22.9 (21.6–24.8)	15.1%	Cross-sectional (Review of service delivery statistics)	**HTS + SRH:** Co-delivery of HTS and cervical cancer screening on-site in SRH/MCH department (where FP also co-located) at 21 government health facilities. Services provided at the same visit and location; enhanced by provider training. “Cervical Cancer Prevention (CECAP) program”

*AGYW, Adolescent girls and young women; ART, antiretroviral therapy; CaCx, cervical cancer; FHOK, Family Health Options Kenya; FLAS, Family Life Association of Eswatini; FP, family planning; FPAM, Family Planning Association of Malawi; FGD, focus group discussion; FSW, female sex worker; HCW, healthcare worker; HTS, HIV testing services; IPPF, International Planned Parenthood Federation; LPPA, Lesotho Planned Parenthood Association; MCH, Mother and Child Health; NAPPA, Namibia Planned Parenthood Association; PMTCT, prevention of mother-to-child HIV transmission; PPAZ, Planned Parenthood Association of Zambia; PrEP, pre-exposure prophylaxis; RHU, Reproductive Health Uganda; SRH, sexual and reproductive health; STI, sexually transmitted infection; VMMC, voluntary male medical circumcision; ZNFPC, Zimbabwe National Family Planning Council*.

* *HIV Prevalence, Total # PLHIV, and % ART coverage among PLHIV (age ≥15) (18)*;

** *Treatment-adjusted HIV prevalence = (Total PLHIV age ≥15 – PLHIV on ART age ≥15)/Total Population 15≥ – PLHIV on ART age ≥15. Data source for total population data (19). Data sources for treatment-adjusted prevalence*:

a *2017 data from UNAIDS and UN POP*,

b *Lesotho (2012 data); Eswatini (2012 data); Botswana (2013 and 2015 data, PLHIV ART coverage for ≥15 not available—thus overall ART coverage was used); Togo (2019 data)*,

c *2017 data from UNAIDS and UN POP*,

d *2008 and 2009 data from UNAIDS and UN POP*,

e *Mozambique, 2009 data*,

f *2019 data*,

g *Tanzania, 2012 data*.

#### HTS Uptake and HIV Positivity in Sub-Saharan Africa

Six programs in sub-Saharan Africa offered HTS within the context of FP service delivery; four providing HTS to women seeking FP services (two in Kenya, one in Uganda, and one in Tanzania) (23–25, 35, 56), one providing HIV counseling in FP clinics and referring women elsewhere for testing in Nigeria (34, 37), and one co-delivering FP and HTS to couples in households in Malawi (40). No programs reported data on linkage to HIV prevention, treatment and care services and other clinical and support services. The Tanzania program targeted female sex workers (FSW), adolescent girls and young women (AGYW), and other “hotspot” populations specifically (“hotspot” was not further defined) (23). Descriptions of programs, including details on the approach to offering HTS in FP/SRH service delivery, are in Table 1 with article extraction sheets in the Supplementary Table.

**Figure 2:**
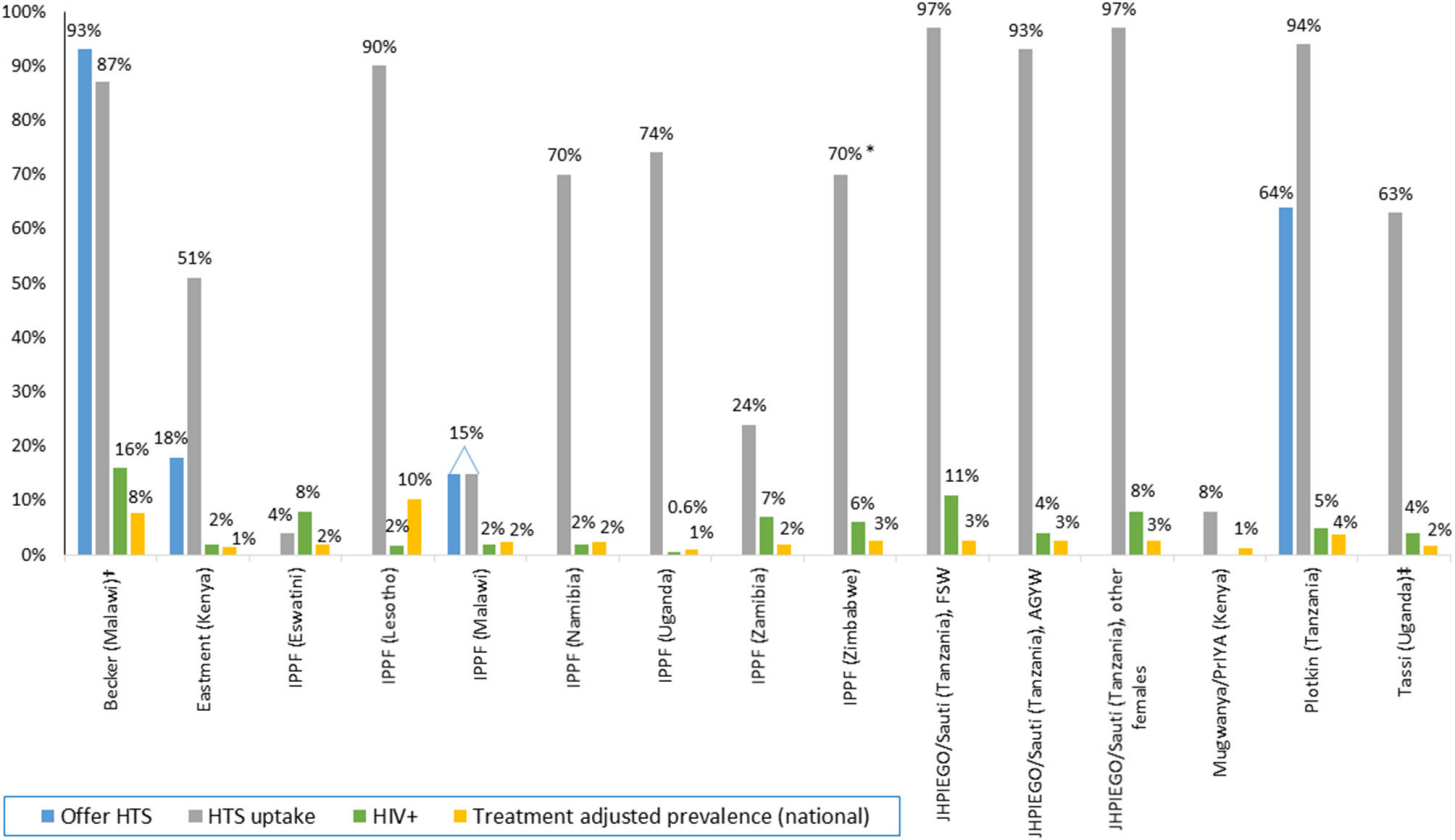
HTS offered, uptake, and positivity among integrated FP programs, by program. Data presented in the figure are rounded to the nearest whole number if it is >1%. Adolescent girls and young women (AGYW); female sex worker (FSW); HIV testing services (HTS); International Planned Parenthood Federation (IPPF). *Range 70–95%. ^†^HTS offer and uptake among male–female couples, HIV positivity among women only. ^‡^Sex not specified.

Only two programs reported data on the proportion offered HTS (Figure 2); all others only provided HTS uptake and/or positivity among those offered HTS. An observational study of FP clinics in Mombasa, Kenya found 59% (23/58) of clinics offered HIV testing to new FP clients, uptake of HTS was 51% (419/814), and 2% were HIV-positive (35). In Malawi, couples received HIV pre-test information together, were individually tested, and then received FP services and condoms in the 20–40 min while awaiting their HIV test results. Couples could also opt to receive only HTS, or only FP services. In this study, 93% of couples (167/180) were offered testing, 87% of couples were tested, and 16% of women were HIV-positive. Over one-quarter (26%, 94/360) of all individuals tested were first-time testers. Prevalence of first-time testing among those tested was 48% among men (*n* = 69) and 17% among women (*n* = 25). Overall, 22.1% (32/145) of couples had at least one positive partner, 12.4% were serodiscordant and 9.7% were concordant positive (40). HTS uptake was also high in the Tanzania study (93–97%), with 11% of FSW, 4% of AGYW, and 8% of “hotspot” populations testing positive (23). Low to moderate HTS uptake was reported in Nigeria (7–14%), Uganda (63%), and in a PrEP implementation program in Kenya (8%) (34, 35, 37, 56). In a larger (*n* = 39 facilities) evaluation conducted over 2 years (2007–2009) in one of the Nigeria programs, uptake of HTS was 7% (2,372 of 32,237 referred received tested). In a separate evaluation of HTS referral models in 40 FP clinics in Nigeria, receipt of an HIV test was 46% higher among women who accepted HTS through the one-stop-shop (“kiosk”) delivery model (100% tested) vs. the referral (“supermarket”) model (77% tested) (34, 37).

Four programs included HTS as part of a package of SRH services; services included varied by program. IPPF member associations offered comprehensive co-delivery of SRH and HIV (HTS, ART, and PrEP) by the same provider in youth-friendly static and mobile clinics (49–55, 57). Some IPPF member associations (Namibia, Zambia) also incorporated the option of referring women to stand-alone HTS services. Seven of eight IPPF countries provided data on HTS; only Malawi reported the frequency of offering HTS (15%). Among individuals offered HTS, uptake was highest in Lesotho (90%), followed by Zimbabwe (80%), Uganda (74%), and Namibia (70%). IPPF member associations in Zambia, Malawi, and Eswatini had low uptake (24, 15, and 4%, respectively). Program managers in Eswatini attributed low HTS uptake to widespread access to HTS elsewhere and large number of clients who were recently tested or women living with HIV already in care, limiting eligibility for HIV testing while seeking FP/SRH services. Despite low HTS uptake, HIV positivity was highest in IPPF programs in Eswatini (8%) and Zambia (7%). HIV positivity was lower in Lesotho, Namibia, and Malawi (all 3%) and Uganda (0.6%).

Another large program delivering bi-directional HIV and SRH services was the SRH & HIV Linkages Project, an interagency collaboration with IPPF, UNAIDS, UNFPA, and WHO; records from this project included in the analysis were from four countries in sub-Saharan Africa (as well as Tunisia and India) (21, 44, 58, 59). The program included different HIV and SRH services and approaches to integration across countries. In Eswatini, enhanced peer mentorship for health care workers was used to provide one-stop-shop co-delivery of services, and data on patient and provider satisfaction were obtained through surveys. Between 2011 and 2013, HTS uptake increased from 0 to 20% (58). Botswana also initially used a one-stop-shop model, offering ART, FP, STI, and cervical cancer screening services in nine sites, incorporating task-shifting and enhanced training though partnerships with NGOs. The proportion of women accessing both HIV and FP services increased from 0% in 2012 to 89% in 2013. In 2015, the Botswana sites offered several approaches to service delivery to both men and women through one-stop-shop models (“kiosk”), delivering services in multiple rooms by different HCW at large clinics (“supermarket”), or referring to different rooms in the same facility with different HCW within a hospital (“mall”). In this integrated program, 63% of male and female clients received both HIV and FP services; 89% of female clients received dual services (21). In Lesotho, an integrated, comprehensive SRH package was offered to a broad range of populations, including men, FSW, adolescents, and gender-based violence survivors. HTS uptake nearly tripled when the program was introduced in 2012 from 3,170 to 8,114 in 2013 based on service delivery statistics (59). In contrast to uptake in Lesotho, provision of integrated services with enhanced training on SRH and HIV within the Togo program led to only 17% HTS uptake among male and female SRH clients (44).

In Nigeria, both one-stop-shop and referral-based approaches to HTS in FP clinics were implemented (34, 37). One year following implementation, 14% (*n* = 32,337) of FP clients received HTS. HTS uptake was higher with the one-stop-shop model than the on-site referral model (100% of individuals accepting HTS at the one-stop-shop completed testing vs. 77% who were referred). Two programs in Tanzania offered HTS in other SRH programs (23, 35). A cervical cancer screening program co-located with FP services offered HTS to 64% (11,819/18,539) of women screened for cervical cancer, of which 94% (11,072/11,819) were tested and 5% were positive (582/11,072) (23). A community-based program (Sauti Project) that targeted FSW, out-of-school adolescent girls and young women (AGYW, ages 15–24), and other female populations in HIV hotspots found high HTS uptake across all populations: 96–97% among FSW, 93–99% among AGYW, and 97–99% among other populations (38). HIV positivity was 5–11% among FSW, 2–4% among AGYW, and 3–8% among other populations (38). International Centre for Reproductive Health (ICRH) also targeted FSW using a night clinic in Mozambique, which included integrated services (30–33). Program evaluations of this model found high client satisfaction (33) and substantial increases on HTS uptake among FSW (30, 32).

#### HTS for Male Partners

Overall, only three programs identified in our review included male partners in their HTS strategies and one reported partner HTS outcomes. The couple study in Malawi found that 45% of male partners were first-time testers (69/145), and HTS uptake was 91% among men who had never tested before (69/76) (40). Male partner HIV status in the PrEP program was also reported; 31% of women did not know their partner's HIV status and 5% of women had an HIV-positive partner (25).

#### Social Harms

The couple study in Malawi was also the only study reporting on social harm resulting from testing, and no social harms were reported (40).

#### Perspectives on Successes and Challenges in Sub-Saharan Africa

Experiences and perceptions of delivering HTS in FP or SRH services were reported by providers and male and female clients from the SRH & HIV Linkages Project in three countries (21, 44, 58), by FSW clients from ICRH in Mozambique (33), and during phone interviews with IPPF program managers from eight countries (49–55, 57) (Table 2). Program managers believed that HTS delivery in an FP/SRH setting was “*easy to do*” [IPPF, Eswatini] at FP initiation and noted many benefits to service integration for clients, including reduced number of trips to health facilities and consultations (42, 44, 58). They also believed that clients were appreciative of delivering integrated service delivery, stating “*People seem to really like when you bring services together*” (IPPF, Malawi). However, they also expressed several concerns about this delivery model. Some program managers acknowledged they had initial fears that adding HTS would overwhelm providers, but IPPF Kenya said concerns were alleviated after the program was implemented. Overall, structural barriers commonly cited by providers and clients include lack of adequate clinic space (54), concerns about longer queues and wait time (42, 44, 58), lack of trained providers (54, 57), and shortage of trained staff (58) and test kits (49, 57).

**Table 2:** Perspectives of clients, providers, and program managers on successes and challenges of integrating HTS in FP programs.

**Theme**	**Sub-theme**	**Representative quotes**
Successes of integrating HTS and FP	*Integration is easy to do*	• “HTS is very easy to do at initiation of FP services.” *Program manager, IPPF Eswatini* (50) • “At first, it was thought [providing HTS] would be a big increase in workload for providers, but that is not an issue anymore [after implementation].” *Program manager, IPPF Kenya* (53)
	*Integration is beneficial*	• Providers believed integration was beneficial for the client. *Providers, SRH & HIV Linkages Project, Eswatini* (58)
	*Clients prefer co-located services*	• “People really like it when you bring services together.” *Program manager, IPPF Malawi* (54) • FGD participants were highly satisfied with integrated services. They experienced positive reception by providers, short waiting times, close proximity, and free services. *FSW, ICRH Mozambique* (33) • Clients preferred to receive SRH and HIV services at the same facility because of reduced travel costs, reduced number of visits, and receipt of complementary and efficient services. Thirty-five percent of SRH clients and 41% of HIV clients preferred to receive both services from the same provider. *Male and female clients, SRH & HIV Linkages Project, Togo* (44) • Eighty-three percent of clients said they were satisfied with service quality. Many (73.6%) preferred SRH and HIV services to be provided at same facility because it reduced travel (57.1%). *Male and female clients, SRH & HIV Linkages Project, Botswana* (21) • Clients reported SRH and HIV integration yielded several benefits, including reduced trips to health facilities, increased service efficiency, and reduced overall health expenditures. *Female clients, SRH & HIV Linkages Project, Eswatini* (58)
	*Integration reduces strain on health providers*	• Providers reported that integrated services preserved nurses' energy with less time moving from one room to another and reduced number of client visits and general consultations. *Providers, SRH & HIV Linkages Project, Botswana* (21)
Challenges of integrating HTS and FP	*Clients do not prefer to test in FP program*	• “When [clients] come for FP, they usually aren't interested in other services. The main thing is that people don't want to test [here]. [Testing] Services are generally readily available and in most cases people have HIV test kits [HIVST].” *Program manager, IPPF Eswatini* (50) • “There is low acceptance [of HTS] because clients say they have already tested or are already HIV+ or on treatment. Sometimes they have other reasons they are not ready to be tested.” *Program manager, IPPF Eswatini* (50)
	*Resources are limited for providing testing in FP settings*	• “Since the shift to new guidelines of providing HTS to high risk populations, the number of HIV test kits has also been reduced in the country [and to the FP facility], but we feel it is an important service to provide in the FP setting.” *Program manager, IPPF Uganda* (57) • “Due to social stigma and criminalization, we need closed, confidential spaces [to provide HTS] for key populations.” *Program manager, IPPF Uganda* (57)
	*Integration strains capacity of health providers*	• “Maybe clients are waiting a little longer [for HTS] because counseling gets extended by 15 minutes or so with the provider.” *Program manager, IPPF Kenya* (53) • “It is overwhelming to provide all integrated services to all clients because it takes time to get all the services and we have limited providers. The best way to address this would be to have a robust outreach team so tasks can be shared.” *Program manager, IPPF Uganda* (57) • Most providers (94.4%) experienced challenges due to increased time spent with clients and many (83.3%) felt an increased workload. *Providers, SRH & HIV Linkages Project, Botswana* (21) • Disadvantages of integration were that service providers would be overwhelmed (35.2%), there would be increased wait times (26.9%), and decreased service quality (10.4%). *Male and female clients, SRH & HIV Linkages Project, Botswana* (21) • Challenges of integration included longer queues, staff shortages, and an increased workload. *Providers, SRH & HIV Linkages Project, Eswatini* (58)

*Direct quotes are only available from IPPF phone interviews conducted for this review. Qualitative reporting from remaining programs was extracted from summary text*.

One site reported no current challenges to providing integrated FP and HTS, but stated, “*Maybe clients are waiting a little longer [for HTS] because counseling gets extended by 15 minutes or so with the provider*” [IPPF, Kenya]. Program managers from other countries believed that a challenge for integration was that, “*when [clients] come for FP, they usually aren't interested in other services*” (IPPF, Eswatini). Some countries said integration can be duplicative because HTS is “*readily available, and in most cases, people already have test kits*” (IPPF, Eswatini) with wider availability of HTS generally, and HIV self-test kits for direct consumer purchase in pharmacies, specifically.

Overwhelmingly, women who received integrated services were very supportive of offering HTS in FP/SRH services whether in the same site or by the same provider. Women appreciated the efficient approach to delivering co-located services, citing fewer trips to the facilities and lower travel and health care costs (33, 42, 44, 58). Women did have varied perceptions about waiting times, with some saying their wait time to receive HTS within FP/SRH was shorter (33), while others said waiting times were longer (42, 44). Some women from a few of the HIV & SRH Linkages Project sites did report that providers seemed overwhelmed or too busy, and the provision of integrated services was perceived by some to be lower quality with less confidentiality (42, 44).

#### Additional Integrated HTS and FP/SRH Programs From Other Regions

Beyond sub-Saharan Africa, the SRH & HIV Linkages Project also integrated SRH and HTS in India, in which 36% of FP clients received HIV counseling and were referred for testing in 2012 (45). From a rapid assessment of the program in India, 48% (9/27) of clients interviewed reported receiving at least one HIV service at the integrated site (45). Additionally, two programs from the USA were included in the analysis (22, 39). An assessment of publicly funded US FP clinics offering HTS and STI services found that 19% of FP clients were tested for HIV (22). HIV testing data from 10 FP clinics serving adolescents and young adults were also collected over a 4-year period in a US study; 86% (34,299/39,698) of clinic patients were tested for HIV. Nearly a quarter (22%, *n* = 7,820) of testers were men and half (51%, 17,585/34,299) were young people (20–24 years). The average number of HIV tests administered at the clinics doubled after implementing routine, opt-out HTS. Overall HIV positivity was 0.3% (88/34,299), 0.8% among men and 0.1% among women.

## Implications

We observed a wide range of studies and programs with variable HTS positivity in this review, reflecting inherent differences in testing uptake, as well as differences in contexts and populations served. These findings also illustrate global shifts in the HIV epidemic due to the scale-up of HIV testing and treatment. In 2019, 87% of people with HIV knew their status and 72% of those who knew their status were on treatment in east and southern Africa (60). As a result of this scale-up and fewer people with HIV who are unaware of their status, despite many countries having high HIV prevalence (>20% in some settings), the national HTS positivity and HIV prevalence among those not on treatment is <5% (61). When we compare HTS positivity from FP clinics within sub-Saharan Africa to other approaches, results are comparable to many facility and community settings (62). Nevertheless, as with all HTS, it is essential to find ways to efficiently target HTS within FP clinics. Strategies are urgently needed to support effective and efficient integration of HTS in FP services so that women with undiagnosed HIV infection or at high ongoing risk can learn their status and benefit from HIV prevention and treatment services.

## Actionable Recommendations

Based on our review, integrating HTS within FP/SRH services was highly variable with limited information about how integration was implemented. We found some examples that suggest that task-shifting and on-site service provision (as opposed to referrals) may be effective approaches to improve co-delivery of services and warrant further exploration. Qualitative data from programs implementing HTS in FP/SRH also highlight structural barriers to consider. Based on these findings, and gaps in the literature that have not previously been reported on, we identify several possible actionable recommendations for consideration.

1. In high HIV burden settings, routine offer of HTS for women seeking FP services may be appropriate, while in medium burden settings, offering HTS may be based on risk or if requested by women. In low burden settings, HTS should not be prioritized in FP clinics unless women are at high risk for HIV, including women who are in serodiscordant couples or are from key populations (people who inject drugs or FSW).
2. Incorporate task-shifting, provision of specific training or supervision for integrating HTS in FP service delivery, peer mentorship for health care workers, or campaigns to support integrated service delivery.
3. Invest in demand creation efforts to reach AGYW and provide a package of SRH services including HTS, FP, and PrEP.
4. Offer HIV self-tests as an alternative approach to overcoming provider concerns and logistical barriers to HTS in FP settings.
5. Develop robust monitoring and evaluation plans to document approaches used to offer HTS within FP services, including the number and type of services offered. Programs should consider monitoring and evaluating a HIV care cascade (offering testing, diagnosis, treatment, and prevention) for women seeking FP/SRH services, similar to the one used in prevention of mother-to-child HIV prevention programs. In addition to measuring the proportion of women who are offered HTS, HTS uptake, and HIV positivity—programs may find it useful to also track linkage to care, treatment, and prevention.
6. Document resources, trainings, and changes in HTS outcomes following program implementation to measure the impact of providing HTS in programs, including details of the service delivery model.
7. Document fidelity of interventions or new programmatic elements introduced to increase HTS to assess validity of these approaches.
8. Apply implementation science frameworks, such as the Consolidated Framework for Implementation Research, to guide efforts to evaluate and optimize design of integrated service delivery models (63).

## Discussion

In our review, few programs (12 overall, 10 in sub-Saharan Africa) had data available on providing HTS in the context of FP and contraception, SRH, or service delivery. HTS uptake was moderate in programs that only reported observational data on efforts to provide HTS with FP/SRH. HTS uptake was higher in some programs where programs included activities such as task-sharing, providing specific training or supervision, peer mentorship for health care workers, or campaigns to support integrated service delivery. In addition, only two programs documented the frequency of offering HTS. Overall HTS uptake in clinics offering FP services in sub-Saharan African to women with considerable HIV risk will likely remain low if HTS is not routinely offered.

Programs used a variety of approaches to offer HTS in FP/SRH services, including co-delivery of services to couples at home, targeting key populations such as FSW, or AGYW, and combining HTS with cervical cancer screening and other SRH programs. While most programs offered one-stop-shop models to deliver services, a few explored models where clients are referred to different providers and rooms within the health care facility. Only one program in Nigeria that offered both one-stop-shop and referral models directly compared HTS outcomes by delivery model, and found HTS uptake was universal (100%) in the one-stop-shop compared to 75% in the referral model.

Our findings concur with those from a prior review on studies with integrated vs. non-integrated approaches to HTS in FP service delivery, which also concluded providing HTS within FP was feasible based on limited evidence (14). In high burden settings, routinely offering HTS within FP service delivery could be a successful strategy to detect HIV among women seeking SRH services (64, 65) and accelerate progress toward 95% of people with HIV knowing their status in the UNAIDS 95-95-95 targets (3). However, the lack of robust, comparative evaluations makes it challenging to determine specific attributes of programs that contribute to success or hinder service delivery. Variability in HTS outcomes across findings may be due to specific approaches used to provide HTS in programs, type and number of SRH services included in the delivery approach, or inconsistencies in program implementation. These inconsistencies could be due to lack of prioritization in providing HTS by health care providers or programs, lack of monitoring and evaluation efforts to measure impact, or perceptions of poor yield/utility. In some programs with low HTS uptake but high HIV positivity, such as Eswatini and Zambia, programs may be differentially offering HTS to high-risk clients, or filling gaps in HTS in settings where HTS is widely available elsewhere. Benefits of integrating HTS into FP service delivery may be attenuated in real-world settings with limited time and training to co-deliver high-quality, rights-based HTS in addition to FP services. Integrated service delivery models may overstretch providers and facilities and increase client waiting time (21, 53, 57, 58). These concerns were articulated in programs that have not yet implemented HTS in FP/SRH or are not consistently implementing HTS, as well as some programs where integration of HTS was underway. However, some providers and program leaders voiced these concerns before the program launched, but later felt that it was feasible to conduct HTS in FP/SRH settings (53). In addition, HIV self-tests may help overcome some barriers to integrating HTS in FP. HIV self-tests have been shown to increase uptake of HIV testing, offer a convenient and confidential testing option, and are recommended by the WHO (66, 67).

A few programs also mentioned that siloed program delivery was another barrier to offering services, with services offered in multiple settings and clients receiving testing in these other settings. Some programs with sub-optimal testing uptake may need to be educated on testing coverage, highlighting gaps in testing, to overcome misconceptions that women do not have a need for testing. In contrast, in highly developed HIV testing programs, testing in FP may not be necessary if test coverage for individuals seeking these services is high through other avenues. If it is desirable to offer integrated HTS and FP service delivery, we will need to invest in coordination between programs to maximize resources.

While many countries have some guidance on offering integrated HTS within SRH, integration of HTS within FP service delivery specifically is only stated in 50% of guidelines of the eight priority country policies we included in this analysis, and lack of clarity on specific services to integrate within guidelines was common (Supplementary Figure) (68–77). The majority of country policies recognize the importance of providing integrated HIV and FP services, but typically related to “reverse” integration, offering FP and contraception in the context of HIV care delivery rather than HTS in FP and contraception services or within the context of MCH (ANC/FP) services. Bi-directional integration of services into both programs is important to improve reproductive health and HIV outcomes.

Programs may consider measuring the effectiveness of a HIV care cascade (offering testing, diagnosis, treatment, and prevention) for women seeking FP/SRH services, similar to the one used in prevention of mother-to-child HIV prevention programs. In addition to measuring the proportion of women who are offered HTS, HTS uptake, and HIV positivity, programs may find it useful to also measure linkage to care, treatment, and prevention. While there is potential that providing HTS alongside FP services has potential to improve both HIV and reproductive health outcomes, none of the studies or programs in our review, or the prior review, reported on these outcomes (14). Furthermore, there is an opportunity to measure benefits of HTS programs by also measuring outcomes for women who test negative, including linkage to HIV prevention services such as PrEP and partner services. In the Kenya PrEP implementation program, offering PrEP in FP clinics led to modest (22%) uptake of PrEP (25).

High HIV incidence was recently reported among women seeking contraception in the ECHO trial in sites in South Africa and Eswatini, but significantly lower in Kenya and Zambia sites, which has led to WHO emphasizing a differentiated approach (78). A different approach, and urgency, to offering HTS within FP/SRH services will be needed depending on local epidemiology and demographic characteristics of women attending services (79).

### Strengths and Limitations

This landscape review had several strengths. We included a diverse range of sources on implementation of HTS in both FP and SRH service delivery, including published articles, gray literature, program reports, and data from qualitative interviews. We included data from high-income settings, low and middle income settings, and focused some aspects of the review on areas of sub-Saharan Africa where HIV prevalence is high. This approach allows us to not only collate lessons learned across settings but also focus in areas with the highest need for integrated HTS and FP/SRH services. Our review is also subject to some limitations. Many details were not provided on program implementation, including training and fidelity of integration approaches. Only one reviewer conducted the primary abstraction, which may have biased inclusion of specific programs included in the review; however, a secondary reviewer did confirm the selection of the programs that were included and contributed to data abstraction. Our search terms were restricted to limit the volume of articles that are on “reverse” integration, which has many more citations but would use a similar search strategy; therefore, we may have missed some articles with this restricted search. We selectively reached out to programs to inquire about availability of data on this topic, but some programs do not have available data while others were excluded from the catchment, which limits the generalizability of our findings.

### Conclusion

Overall, there is limited evidence available to fully evaluate feasibility and efficiency of providing HTS in FP services or SRH settings. Though infrequently reported, we know that these data exist in some countries based on instruction in national policies (76). Future efforts should focus on better outcome ascertainment and characterization of the context surrounding provision of HTS within FP/SRH service delivery. Investments to support integration efforts, including time and training to deliver high-quality services, are needed to ensure high HTS coverage and prevent MTCT.

## Author Contributions

AD and CJ developed and designed this landscape analysis, with engagement from MG, JK, and RB. AD and CQ collected, analyzed, and interpreted the evidence for the review. AD drafted the manuscript. All authors provided critical revision and final approval of the article.

## Conflict of Interest

The authors declare that the research was conducted in the absence of any commercial or financial relationships that could be construed as a potential conflict of interest.
