# Supplementary material for: A Landscape Analysis of Offering HIV Testing Services Within Family Planning Service Delivery
Source: Front Reprod Health. 2021 May 26;3:657728. doi: 10.3389/frph.2021.657728 (PMC9580748; doi:10.3389/frph.2021.657728)
Supplement: Supplementary file 1 [file Table_1.DOCX]

Supplementary Figure. Countries with national policies on HTS and FP integration

**
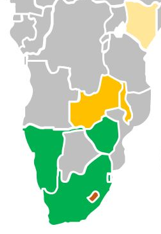
**

*

*

**

**

**RECOMMENDATIONS**

HTS in FP *(*FP GL only, **HTS GL only)*

PITC (not specific to FP)

General (not specific to HTS or FP)

No guidance

Not reviewed

Caption: family planning (FP); guidelines (GL); HIV testing services (HTS); PITC (provider initiated testing and counseling)
